# *In Vitro* Effects of Herbicides and Insecticides on Human Breast Cells

**DOI:** 10.5402/2012/232461

**Published:** 2012-10-14

**Authors:** Jessica D. Rich, Seth M. Gabriel, Jennifer R. Schultz-Norton

**Affiliations:** Department of Biology, Millikin University, 1184 West Main Street, Decatur, IL 62522, USA

## Abstract

Numerous studies have indicated that the pesticides and herbicides used in agricultural processes in the United States and Europe may have detrimental effects upon human health. Many of these compounds have been indicated as potential endocrine and reproductive disruptors, although the studies have examined supraphysiological levels well above the US EPA safe levels for drinking water and have often examined these effects in “model” cell lines such as Chinese hamster ovary cells. We have now examined the cytotoxicity of more environmentally relevant concentrations of four herbicides, acetochlor, atrazine, cyanazine, and simazine, and two insecticides, chlorpyrifos and resmethrin, in three human breast cell lines. Interestingly, cytotoxicity was not observed in the estrogen-dependent MCF-7 mammary epithelial carcinoma cells; rather increases in cell viability were seen for some of the compounds at select concentrations. These results vary greatly from what was observed in the estrogen independent MDA-MB-231 breast cancer cells and the non-cancerous MCF-10A breast cells. This gives insight into how different tumors may respond to pesticide exposure and allows us to make more accurate conclusions about the potential cytotoxicity or, at times, stimulatory actions of these pesticides.

## 1. Introduction

In recent years, correlative studies have indicated that the pesticides and herbicides used routinely in crop production may in fact have detrimental effects upon human health. The Crop Protection Research Institute lists 24 fungicides, 41 insecticides, and 75 herbicides that are commonly used in Illinois. As of 2002, over 27 million pounds of herbicide and 1.3 million pounds of insecticide were applied in Illinois per year in corn production alone [1]. These levels, when calculated regionally, show very high levels of pesticide application in the Lake Decatur watershed area, which supplies drinking water to the campus where this research is based (Table 1). This includes more than 100,000 pounds of atrazine, which is thought to have detrimental effects on reproductive development and increases aromatase expression in ovarian cancer, adrenocortical carcinoma, and placental choriocarcinoma cells and granulosa-lutein cell cultures [2–6]. These compounds have also been shown to be endocrine disruptors by altering hormone metabolism [7]. These chemicals may leach into our groundwater and thus increase our exposure, and levels in excess of the US EPA Maximum Contamination Level (MCL) have been previously observed [6, 8–11]. 

The following compounds were selected for this study due to their toxicity and use in central Illinois crop production. Atrazine, 2-chloro-4-ethylamino-6-isopropylamino-s-triazine, is one of the most widely used herbicides in the United States. However, it is considered to be a common terrestrial and aquatic contaminant [12]. Although it is not generally considered to cause adverse reproductive effects, and is not teratogenic or mutagenic, it has been shown to cause the development of mammary tumors in rats who were exposed to atrazine over lifetime administration [13]. It has also been shown to disrupt reproductive tract development in aquatic organisms [14, 15]. Cyanazine, 2-(4-chloro-6-ethylamino-1,3,5-triazin-2-ylamino)-2-methylpropionitrile, is a herbicide that is considered moderately toxic. Exposure resulted in a decrease in maternal body weight gain in rats and decreased fetal viability in rabbits [16]. Although not considered carcinogenic, it is highly teratogenic and is also known to cause depression of the nervous system [17]. Simazine, 6-chloro-N2,N4-diethyl-1,3,5-triazine-2,4-diamine, is considered slightly nontoxic, although high rates of fetotoxicity and decreased birth weight after high exposure were observed in rabbits [18]. It has been shown to exhibit some mutagenicity in human lung cell cultures and caused both thyroid and mammary tumors in rats [13, 19]. Acetochlor, 2-chloro-N-(ethoxymethyl)-N-(2-ethyl-6-methylphenyl)acetamide, is commonly used as a component in Guardian, Harness, Relay, Surpass, and Top-Hand brands [20] and is classified as highly toxic. It is a restricted use pesticide although it is currently labeled by the EPA only as a potential human carcinogen. Studies performed on dogs have determined that exposure to acetochlor results in a decrease in body weight, testicular atrophy, and increased adrenal weight [13]. In rats, acetochlor exposure has been found to decrease litter size and increase both prostate and thyroid weight. Additionally, acetochlor induces a weak DNA repair response and decreased pregnancy rates in rats [21]. It has been shown to increase the expression of the thyroid hormone *β* receptors in tadpoles [22, 23]. 

In addition to the above-mentioned herbicides, two insecticides were also selected for study. Chlorpyrifos, *O,O*-diethyl *O*-3,5,6-trichloro-2-pyridyl phosphorothioate, is an organophosphate insecticide whose function is to inhibit acetylcholinesterase and can be toxic to the human nervous system [24]. While chlorpyrifos does not appear to be teratogenic, a slight increase in the number of pup deaths was observed after oral chlorpyrifos administration to the dams [13]. Additionally, chlorpyrifos has been shown to inhibit p450 enzymes responsible for estrogen and testosterone metabolism [25, 26]. Resmethrin, 5-benzyl-3-furylmethyl (1RS)-cis,trans-2,2-dimethyl-3-(2-methylprop-1-enyl)cyclopropanecarboxylate, is a synthetic pyrethroid insecticide used for control of flying and crawling insects and acts by disrupting sodium channels in nerves. Although it is considered nontoxic when ingested, a slight increase was noted in the number of stillbirth pups in a multigenerational study in rats when exposed to high levels of resmethrin [27].

Some of these compounds like atrazine may act as endocrine disruptors by binding to nuclear hormone receptors. While binding affinities of these compounds to the estrogen receptor may be low, proliferative effects have been observed when atrazine interacts with the estrogen-binding protein GPR30 [28]. However, the functional consequences of these types of interactions have not been examined in detail. A recent publication by The Endocrine Society states that an endocrine-disrupting substance is any chemical which alters hormonal and homeostatic systems [29]. Thus, it becomes pertinent to examine the molecular and physiological effects of these compounds. While this paper in particular does not focus upon the endocrine-disrupting effects of pesticides, it does compare the cytotoxic potential to that of 17*β*-estradiol, which is frequently used as a positive control in studying potentially estrogenic, endocrine-disrupting compounds, in cells that should and should not respond to estrogenic compounds. 

While other studies have begun to decipher the potential cytotoxicity of a variety of pesticides [20], Rayburn et al. in particular focused upon supraphysiological and supraenvironmental concentrations of atrazine and acetochlor, with levels up to 800,000-fold higher than the EPA safe level for atrazine in drinking water [10]. Although it is important to look at the effect of a large dose of these compounds, it is more physiologically relevant to examine the effects of levels of these pesticides that are present in the environment. To begin analyzing the potential effects that these pesticides may have upon cancerous cells, we have now analyzed the cytotoxicity of physiological concentrations of six pesticides in three different breast cell lines commonly utilized in breast cancer research and compared their activity to that of 17*β*-estradiol. One of these cell lines, MCF-7, has been widely used to examine estrogen-dependent transactivation [29–31]. MDA-MB-231 cells are another mammary epithelial carcinoma but do not express the estrogen receptor and thus are not considered estrogen-dependent [32]. A third cell line, MCF-10A, was developed from normal mammary epithelial cells and is not generally considered to be estrogen-responsive [33, 34]. In choosing these three cell lines as model systems, we can begin to compare the potentially cytotoxic or stimulatory effects of physiologically relevant concentrations of these pesticides on both estrogen-dependent and independent breast cancers as well as a normal, non-cancerous cell line. This will assist us in answering the question of whether exposure to these compounds is potentially hazardous for women with breast cancer.

## 2. Materials and Methods

### 2.1. Chemicals

The following chemicals were obtained from Sigma-Aldrich (St. Louis, MO): dimethylsulfoxide (DMSO), Minimum Essential Medium Eagle (MEM), acetochlor (CAS 34256-82-1), atrazine (CAS 1912-24-9), chlorpyriphos (CAS 2921-88-2), cyanazine (CAS 21725-46-2), resmethrin (CAS 10453-86-8), and simazine (CAS 122-34-9). All compounds were analytical grade quality with a minimum purity of 94%. 17*β*-estradiol was purchased from Cayman Chemical Company (Ann Arbor, MI). MEM Richter's Modification was obtained from Hyclone (Logan, UT). Leibovitz's L-15 Medium, MEGS supplement, epidermal growth factor, horse serum, and DMEM-F12 media were purchased from Gibco (Grand Island, NY). Calf serum and fetal bovine serum were purchased from PAA Laboratories (Dartmouth, MA). Penicillin-Streptomycin solution and MEM without phenol red were purchased from Cellgro (Manassas, VA). Gentamycin sulfate was obtained from Teknova (Hollister, CA). Cholera toxin was purchased from Sigma-Aldrich (St. Louis, MO). Crystal violet was purchased from Allied Chemical (New York, NY). MCF-7, MDA-MB-231, and MCF-10A cells were obtained from Dr. Ann M. Nardulli (University of Illinois, Urbana, IL).

### 2.2. Cell Maintenance

MDA-MB-231 cells were maintained in a closed-flask system, while MCF-7 and MCF-10A cells were maintained in a 5% CO_2_ humidified environment. MCF-7 mammary epithelial carcinoma cells were maintained in phenol red-containing MEM, supplemented with 5% calf serum and antibiotics (50 IU/mL penicillin, 50 *μ*g/mL streptomycin, and 5 *μ*g/mL gentamycin sulfate). MDA-MB-231 mammary epithelial carcinoma cells were maintained in Leibovitz's L-15 medium supplemented with 10% heat-inactivated fetal bovine serum and 5 *μ*g/mL gentamycin. MCF-10A cells were maintained in DMEM-F12 media that supplemented with 5% horse serum, 0.1 *μ*g/mL cholera toxin, 40 ng/mL epidermal growth factor, and MEGS supplement. All cells were split via trypsinization at 80% confluency.

### 2.3. Cytotoxicity Assays

Cytotoxicity assays were performed in accordance with the procedures reported in Rayburn et al. [20]. MCF-10A cells were maintained in DMEM-F12 media supplemented 5% horse serum, 0.1 *μ*g/mL cholera toxin, 40 ng/mL epidermal growth factor, and MEGS supplement for the entirety of the experiment. Twenty-four hours post-split, MDA-MB-231 cells were transferred to MEM supplemented with 5% charcoal dextran-treated calf serum (CDCS) and antibiotics. Forty-eight hours post-split, media for MCF-7 and MDA-MB-231 cells was replaced with phenol red-free MEM supplemented with 5% CDCS and antibiotics and maintained in an open-flask system in the presence of 5% CO_2_. 

Cells were seeded seventy-two hours post-split at 6000 cells per well in a 96-well plate in phenol-red free MEM supplemented with 5% CDCS and antibiotics (for MCF-7 and MDA-MB-231 cells) or DMEM-F12 media supplemented with 5% horse serum, 0.1 *μ*g/mL cholera toxin, 40 ng/mL epidermal growth factor, and MEGS supplement (for MCF-10A cells). After twenty-four hours, the cells were treated with 10, 100, 1000, or 10000 nM 10 *μ*M 17*β*-estradiol or pesticide or with DMSO control. Treatment compounds were resuspended in DMSO at a concentration of 10 mM and were diluted further with DMSO to maintain constant volumes of DMSO in all experiments, regardless of compound concentration. The final volume of DMSO in media was kept at 0.1%. After 48 hours, live cells were fixed with 50% methanol and stained with crystal violet in 10% methanol. Stained cells were lysed and the crystal violet resuspended in 1% SDS. The amount of stain absorbed by each well was quantified using a microplate reader (Anthos htII) at 540 nM. The amount of stain was reflective of living cells. Treatments were carried out in triplicate, and three independent experiments were performed per pesticide in each cell line. Results are presented as percent of live cells compared to the DMSO control, set at 100% confluency. Significant deviations from the control for each cell line were calculated by student's *t*-test (two-tailed, assuming unequal variances). Overall combinatorial analysis of the effects of cell type, compound, and concentration were computed using a univariate general linear model and one-way ANOVA with homogeneity tests and LSD post hoc (IBM SPSS Statistics, IBM Corp., Armonk, NY).

## 3. Results

**Figure 1:**
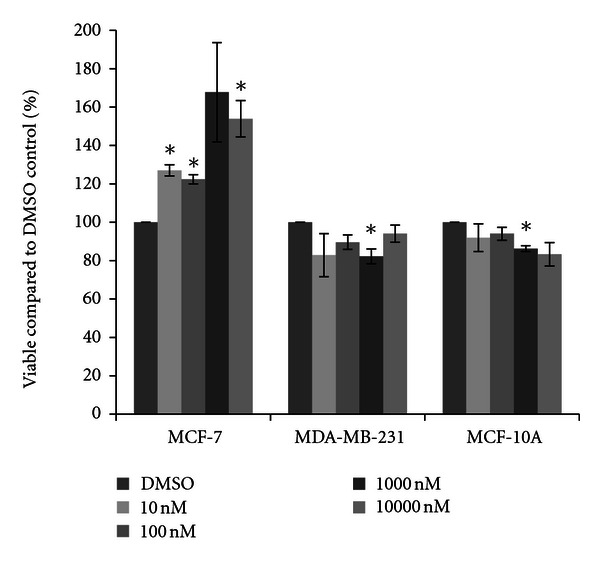
Cytotoxicity of 17*β*-estradiol. MCF-7 and MDA-MB-231 mammary epithelial carcinoma and MCF-10A mammary epithelial cells were plated in a 96-well plate at 6000 cells per well and treated with 10 nM to 10000 nM 17*β*-estradiol or DMSO vehicle control for 48 hours. Live cells were stained with crystal violet and the amount of dye absorbed was quantified by a microplate reader at 450 nm. Results are shown as percentage of live cells compared to DMSO control set at 100 percent. Significant variation and trends of variation from the control were calculated by student's *t*-test (a*, *P* < 0.05).

To examine the cytotoxic effects of physiological and environmental concentrations of a variety of pesticides used in Illinois crop production, three human mammary cell lines were selected: MCF-7 and MDA-MB-231 mammary epithelial carcinoma cells and MCF-10A mammary epithelial cells. These cell lines have been used extensively by the authors for studies examining changes in transcriptional activity by estrogenic compounds ([37–39] and unpublished data). Estrogenic compounds such as 17*β*-estradiol typically have trophic effects upon carcinoma cells which contain the estrogen receptor, such as the MCF-7 cell line, yet do not have effects on cells that do not express the estrogen receptor, such as the MDA-MB-231 cells, so this compound was added as a control. Each cell line was treated with physiologically relevant concentrations of 17*β*-estradiol, ranging from 10 nM to 10000 nM (10 *μ*M), for 48 hours and living cells were quantified using the cytotoxicity method as previously published [20]. As expected, almost all concentrations of 17*β*-estradiol significantly increased cell concentrations within the MCF-7 cell line above that of the DMSO vehicle control (Figure 1). Trends of increased cell number were also observed for the 1000 nM concentration, although these did not statistically differ from the control due to large variation between experiments. In the other two cell lines, 17*β*-estradiol did not increase cell growth and instead slightly reduced cell viability. Significant reduction of cell viability was observed with 1000 nM 17*β*-estradiol for both the MDA-MB-231 and MCF-10A cells, with up to a seventeen percent reduction in the number of live cells present.

**Figure 2:**
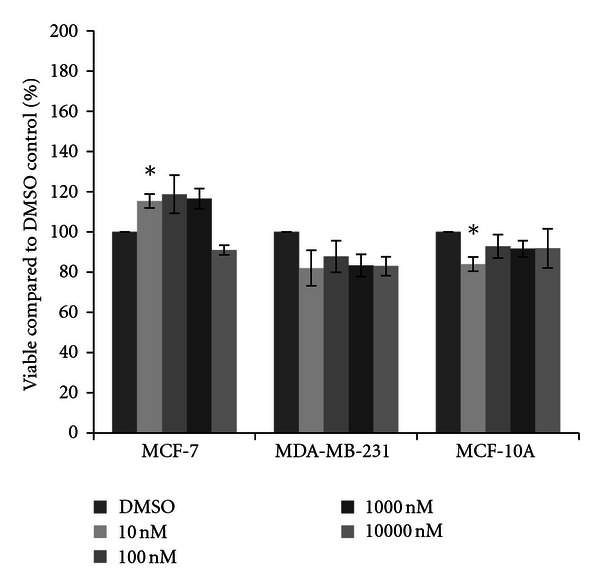
Cytotoxicity of acetochlor. MCF-7 and MDA-MB-231 mammary epithelial carcinoma and MCF-10A mammary epithelial cells were plated in a 96-well plate at 6000 cells per well and treated with 10 nM to 10000 nM acetochor or DMSO vehicle control for 48 hours. Live cells were stained with crystal violet and the amount of dye absorbed was quantified by a microplate reader at 450 nm. Results are shown as percentage of live cells compared to DMSO control set at 100 percent. Significant variation and trends of variation from the control were calculated by student's *t*-test (a*, *P* < 0.05).

**Figure 3:**
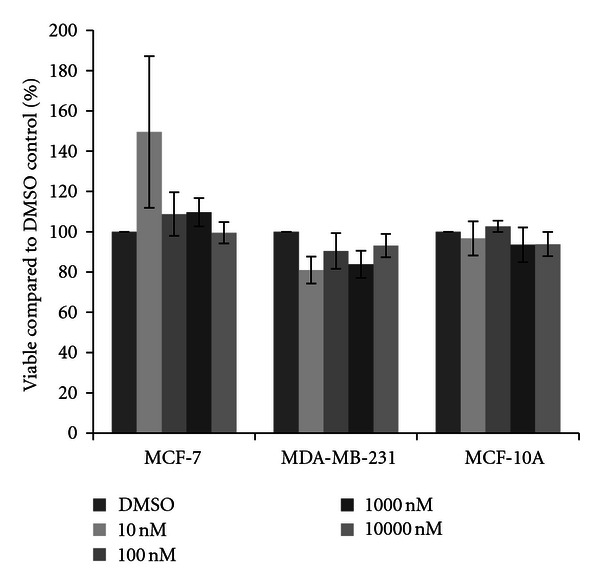
Cytotoxicity of atrazine. MCF-7 and MDA-MB-231 mammary epithelial carcinoma and MCF-10A mammary epithelial cells were plated in a 96-well plate at 6000 cells per well and treated with 10 nM to 10000 nM atrazine or DMSO vehicle control for 48 hours. Live cells were stained with crystal violet and the amount of dye absorbed was quantified by a microplate reader at 450 nm. Results are shown as percentage of live cells compared to DMSO control set at 100 percent. Significant variation and trends of variation from the control were calculated by student's *t*-test (a*, *P* < 0.05).

**Figure 4:**
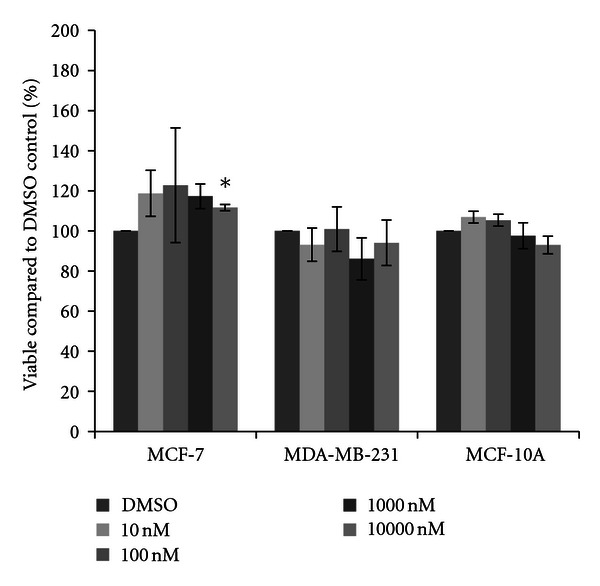
Cytotoxicity of cyanazine. MCF-7 and MDA-MB-231 mammary epithelial carcinoma and MCF-10A mammary epithelial cells were plated in a 96-well plate at 6000 cells per well and treated with 10 nM to 10000 nM cyanazine or DMSO vehicle control for 48 hours. Live cells were stained with crystal violet and the amount of dye absorbed was quantified by a microplate reader at 450 nm. Results are shown as percentage of live cells compared to DMSO control set at 100 percent. Significant variation and trends of variation from the control were calculated by student's *t*-test (a*, *P* < 0.05).

**Figure 5:**
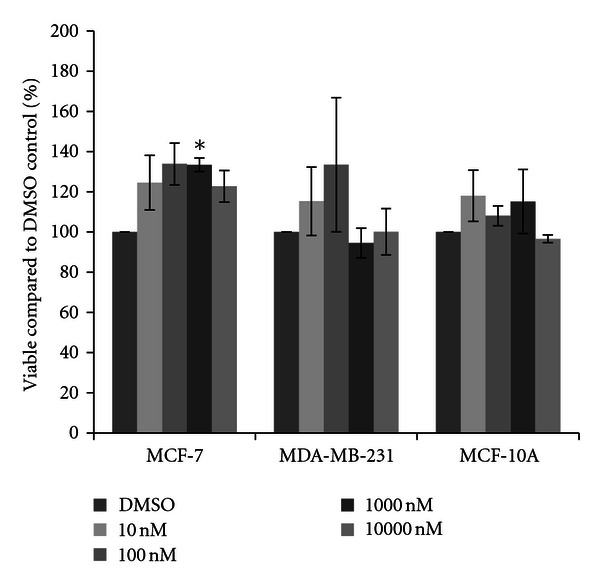
Cytotoxicity of simazine. MCF-7 and MDA-MB-231 mammary epithelial carcinoma and MCF-10A mammary epithelial cells were plated in a 96-well plate at 6000 cells per well and treated with 10 nM to 10000 nM simazine or DMSO vehicle control for 48 hours. Live cells were stained with crystal violet and the amount of dye absorbed was quantified by a microplate reader at 450 nm. Results are shown as percentage of live cells compared to DMSO control set at 100 percent. Significant variation and trends of variation from the control were calculated by student's *t*-test (a*, *P* < 0.05).

 Cells were next treated with one of four herbicides. For acetochlor, little variation from the DMSO vehicle control was observed in MCF-7 cells, although a significant fifteen percent increase in cell density was observed at the 10 nM concentration of acetochlor (Figure 2). In MDA-MB-231 cells, trends for an average sixteen percent decrease in cell density were recorded, although the decreases were not statistically significant. In MCF-10A cells, similar reductions were observed, with significant decreases at 10 nM acetochlor. Three triazine herbicides were also examined. Similar to the effects seen with acetochlor, atrazine and cyanazine failed to dramatically change cell density in all three cell lines (Figures 3 and 4), although there was an indication of possible increase with 10 nM atrazine and a slight yet significant eleven percent increase with 10000 nM cyanazine in MCF-7 cells. Interestingly, a trend for over thirty percent increased cell viability was observed with simazine in both the MCF-7 and MDA-MB-231 cells (Figure 5). Significant increases in cell density were only observed for the 1000 nM simazine treatment in MCF-7 cells although a trend was observed for more cell viability at all concentrations. No significant changes were observed for the MCF-10A cells for any of the triazines. 

**Figure 6:**
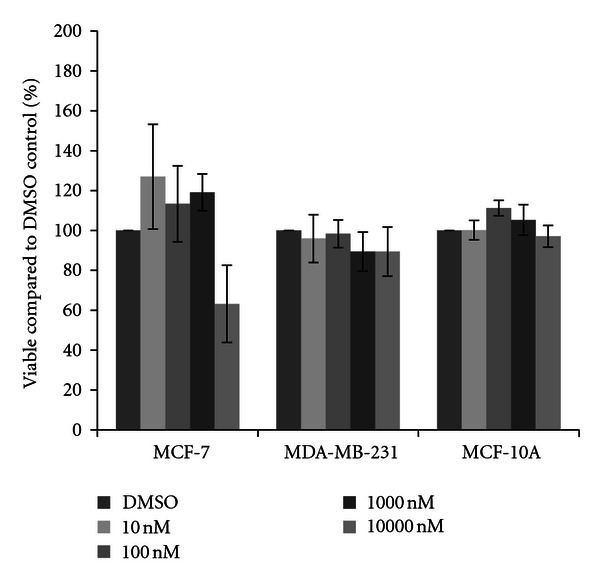
Cytotoxicity of chlorpyrifos. MCF-7 and MDA-MB-231 mammary epithelial carcinoma and MCF-10A mammary epithelial cells were plated in a 96-well plate at 6000 cells per well and treated with 10 nM to 10000 nM chlorpyrifos or DMSO vehicle control for 48 hours. Live cells were stained with crystal violet and the amount of dye absorbed was quantified by a microplate reader at 450 nm. Results are shown as percentage of live cells compared to DMSO control set at 100 percent. Significant variation and trends of variation from the control were calculated by student's *t*-test (a*, *P* < 0.05).

**Figure 7:**
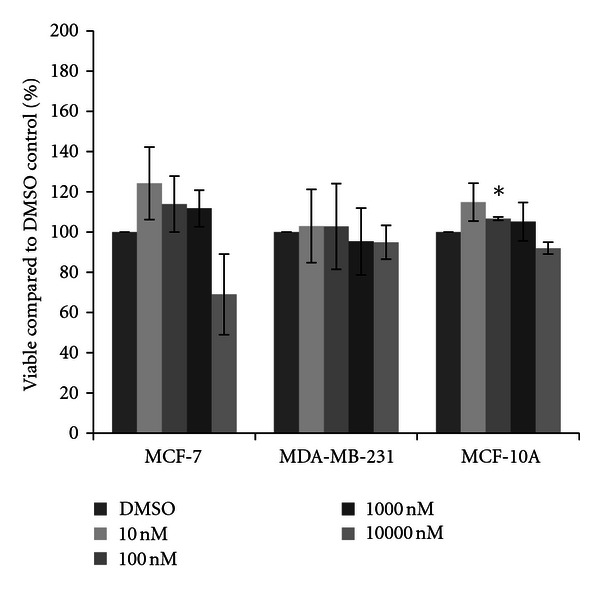
Cytotoxicity of resmethrin. MCF-7 and MDA-MB-231 mammary epithelial carcinoma and MCF-10A mammary epithelial cells were plated in a 96-well plate at 6000 cells per well and treated with 10 nM to 10000 nM resmethrin or DMSO vehicle control for 48 hours. Live cells were stained with crystal violet and the amount of dye absorbed was quantified by a microplate reader at 450 nm. Results are shown as percentage of live cells compared to DMSO control set at 100 percent. Significant variation and trends of variation from the control were calculated by student's *t*-test (a*, *P* < 0.05).

Two insecticides were also checked for cytotoxicity at the same physiological concentrations. Chlorpyrifos did not appear to affect either the MDA-MB-231 or MCF-10A cells, while some alterations were observed in the MCF-7 cells (Figure 6). Although not statistically significant, the 10000 nM concentration of chlorpyrifos showed a marked thirty-seven percent reduction in cell viability. Resmethrin showed a similar pattern in all three cell lines, where the highest concentration of the insecticide showed reduction of cell viability in MCF-7 cells yet no differences were observed in MDA-MB-231 cells (Figure 7). A very slight, yet statistically significant increase in cell density was observed in MCF-10A cells at the 100 nM concentration (six percent increase). 

**Figure 8:**
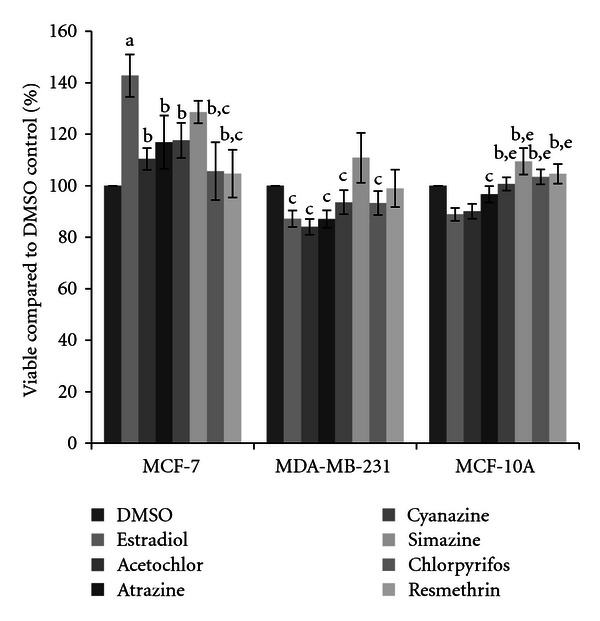
Cytotoxicity of pesticides in three breast cell lines. MCF-7 and MDA-MB-231 mammary epithelial carcinoma and MCF-10A mammary epithelial cells were plated in a 96-well plate at 6000 cells per well and treated with pesticide or DMSO vehicle control for 48 hours. Live cells were stained with crystal violet and the amount of dye absorbed was quantified by a microplate reader at 450 nm. Results are shown as percentage of live cells compared to DMSO control set at 100 percent, with no regard to the concentration of pesticide used. Significant differences in cell viability were calculated by one-way ANOVA with homogeneity tests and LSD post hoc using IBM SPSS Statistics. Pairwise comparisons are shown, with the following significant variations (*P* < 0.05) labeled: (a) differs from DMSO; (b) differs from 17*β*-estradiol; (c) differs from simazine; (d) differs from cyanazine; (e) differs from acetochlor.

Since we had not observed many statistically significant differences when compared to the negative control via student's *t*-test, we took a broader look at the effects of the pesticides using cross-comparisons through univariate ANOVA for the effect of cell type, pesticide, and concentration individually, as well as all combinatorial effects. Each variable was found to be very statistically significant (*P* ≤ 0.003), as was the combined effect of compound and cell (overall *P* ≤ 0.001) and the effect of concentration and cell (*P* = 0.04). The post-hoc comparison showed that there was a statistically significant difference in the overall viability observed in the MCF-7 cells compared to the other cell lines (*P* < 0.000), with a twenty-three percent higher viability in the MCF-7 cells compared to the MDA-MB-231 cells and an eighteen percent higher viability compared to the MCF-10A cells. The difference between the other two cell lines was not statistically significant. As the univariate ANOVA indicated that there was a significant effect of pesticide dependent upon cell type, a one-way ANOVA was performed, allowing for cross-comparisons between pesticides within each cell type and disregarding concentration differences. The results of these comparisons, showing statistical differences in viability between compounds, are shown in Figure 8.

## 4. Discussion

We have now examined the cytotoxicity of a variety of pesticides commonly used in Illinois agricultural applications. What sets apart this research is two factors: first, we chose to examine a variety of human cell lines that are commonly used for endocrine-based research, rather than focusing on only one cell line or on a nonhuman cell line as has previously been done. This is important as although there are strong similarities in mammalian endocrine and reproductive systems, the cell types and physiological responses do vary depending upon species. Second, we have examined low, environmentally, and physiologically relevant concentrations of these chemicals that might be found in the groundwater systems surrounding agricultural application [9, 40]. Interestingly, although their chemical structures are very similar, differences were observed in how the triazine herbicides atrazine, cyanazine, and simazine affected the breast cell growth.

As expected, we did see increases in cell viability in the MCF-7 mammary epithelial cancer cells after treatment with 17*β*-estradiol (Figure 1). This was not observed in the other two breast cell lines; in fact 17*β*-estradiol limited cell viability to a minor extent. However, only the MCF-7 cells express the estrogen receptor [32, 34, 41]; thus these should be the only cells to show increases in viability. Interestingly, cytotoxicity was not observed in the MCF-7 cell line in the presence of four herbicides. Rather, in a few select circumstances increases in cell viability were seen, and trends of increases were observed for acetochlor, atrazine, cyanazine, and simazine. No significant increases were observed with either insecticide (chlorpyrifos or resmethrin). When significant increases after herbicide treatment were observed, a ten to twenty percent increase in cell viability compared to the vehicle control was present (Figure 8). This is not much less than the expected increase in cell count seen with 17*β*-estradiol, a known trophic factor for estrogen-receptor positive breast cancer cells [29–31]. This is of concern as this is indicative of these pesticides possibly increasing cancerous cell growth. This directly supports correlative studies in both the United States and in Europe which have indicated that exposure to pesticides in rural communities may result in higher levels of breast cancer than are recorded in more urban settings [42, 43]. 

For most of the compounds examined, the MDA-MB-231 and MCF-10A cells showed trends toward a decrease in cell viability (Figure 8), although these differences were minor and did not vary significantly from the control. Instead, they did vary significantly from simazine, which trended towards an increase in cell viability, indicating that this herbicide reacted very differently in the cells than any other compound tested. While the US EPA continues to maintain that simazine is not a carcinogenic compound, derivatives of simazine have been shown to disrupt the hypothalamic-pituitary-gonadal axis which, in turn, can alter the growth of mammary tissue. Levels that exceed the recommended drinking water level of comparison for infants and children have been detected in the community water system in several communities within southern central Illinois, the closest being only 60 miles south of where this research was conducted [44, 45]. The health status of those in agricultural communities should be closely monitored as the research presented herein indicates that simazine could potentially affect mammary epithelial cells.

Although the levels of the pesticides used in this study are just outside the EPA recommended MCL levels (Table 2), they fall within physiological ranges for hormone studies (between 10 and 100 nM). As many cytotoxicity studies examine *μ*M to mM concentrations of toxicants, it is disturbing that such low levels of these compounds (10 nM in some cases) could cause changes in cell viability. While the EPA has stated that some of these compounds are not carcinogenic, studies have shown dramatic reproductive responses such as alterations in testicular and prostate size and reduction in litter sizes [13, 21]. It is of great concern that in many reports, triazine herbicides such as atrazine have been shown to have detrimental reproductive and endocrine-disrupting effects and have even been indicated in altered fertility rates in humans [46–50], and yet the overall status of these compounds is referred to as “inconclusive” with regard to their toxicity [51]. Repeatedly, these compounds have been found contaminating surface water in levels that exceed the EPA MCL levels, and this contamination extends beyond the United States to Europe where levels of atrazine and acetochlor an order of magnitude higher than the EU allowed environmental levels have also been reported [9, 10, 40]. Clearly, more studies need to be done in human-based systems. Unfortunately, experimentation in humans is not feasible thus we must pursue other methodologies, especially the utilization of human cell lines, to mimic the response in human tissues as much as possible. 

One of the striking results from this research is the strong difference observed in the estrogen-receptor positive and the estrogen-receptor negative breast cells, while there is little to no difference between the estrogen-receptor negative cancerous and non-cancerous cells. In the presence of the estrogen receptor, it appears that some of these compounds are stimulatory in nature and enhance cell growth, yet in the absence of the estrogen receptor they may be activating apoptotic pathways and thereby increasing cell death. It would be interesting to do an in depth epidemiological study examining cancer incidence in rural communities and look at the application areas for these pesticides to determine if there is any correlation in the rate of breast cancer incidence, as well as the types of breast cancers identified (estrogen-receptor positive versus estrogen-receptor negative). Some correlative studies have been done, but the hormone responsiveness of the tumors is not often examined [42, 52, 53]. This is something to take note of, as there are many differences in the ways that estrogen-receptor positive cancers are treated clinically compared to the estrogen-receptor negative tumors [54–56]. We hope that this research highlights the need for more research in human-based systems and that care must be taken in determining the model system used in toxicological studies.

## Figures and Tables

**Table 1:** Application levels of pesticides in Illinois. Levels of acetochlor, atrazine, cyanazine, simazine, and chlorpyrifos that were applied agriculturally in 1992, 1997, and 2002 were examined. Levels for nation-wide application and Illinois-only application are reported in pounds of pesticide. The average pounds per square mile were calculated for the Illinois data and this was used to calculate the amount of pesticide applied in the Lake Decatur watershed, a 925 square mile area. Application data was obtained from the National Pesticide Use Database [1].

	1992	1997	2002
Acetochlor			
lbs applied in USA	n/a	32591175	36160962
lbs applied in Illinois	n/a	5333375	6479022
lbs applied in Lake Decatur Watershed	n/a	88741	107803

Atrazine			
lbs applied in USA	72315295	74560407	76914999
lbs applied in Illinois	11635842	10908509	14120444
lbs applied in Lake Decatur Watershed	193606	181504	234947

Chlorpyrifos			
lbs applied in USA	14764535	13463879	8481225
lbs applied in Illinois	1456351	960586	468103
lbs applied in Lake Decatur Watershed	24232	15983	7789

Cyanazine			
lbs applied in USA	32189859	20233056	n/a
lbs applied in Illinois	6566591	3663583	n/a
lbs applied in Lake Decatur Watershed	109260	60958	n/a

Simazine			
lbs applied in USA	3978487	5224439	4792495
lbs applied in Illinois	248417	302169	565446
lbs applied in Lake Decatur Watershed	4133	5028	9408

**Table 2:** Pesticide use levels for this study compared to the USEPA limits. The upper and lower limits of pesticides used in this study are reported in *μ*g/L. The USEPA maximum contamination levels (MCL) for drinking water and lifetime health advisory limits are also reported. “ND” indicates that the data was unavailable or a value has not been set by the EPA for that compound. MCL and health advisory data obtained from the USEPA [35, 36].

	Minimum (*μ*g/L)	Maximum (*μ*g/L)	MCL (*μ*g/L)	Health advisory, lifetime (*μ*g/L)
Acetochlor	2.697	2697	2	ND
Atrazine	2.157	2157	3	200
Chlorpyrifos	3.506	3506	1	20
Cyanazine	2.407	2407	1	1
Resmethrin	3.385	3385	ND	ND
Simazine	2.017	2017	4	4
